# Using a Data-Constrained Model of Home Range Establishment to Predict Abundance in Spatially Heterogeneous Habitats

**DOI:** 10.1371/journal.pone.0040599

**Published:** 2012-07-16

**Authors:** Mark C. Vanderwel, Jay R. Malcolm, John P. Caspersen

**Affiliations:** 1 Faculty of Forestry, University of Toronto, Toronto, Canada; 2 Computational Ecology and Environmental Science Group, Microsoft Research, Cambridge, United Kingdom; University of Alberta, Canada

## Abstract

Mechanistic modelling approaches that explicitly translate from individual-scale resource selection to the distribution and abundance of a larger population may be better suited to predicting responses to spatially heterogeneous habitat alteration than commonly-used regression models. We developed an individual-based model of home range establishment that, given a mapped distribution of local habitat values, estimates species abundance by simulating the number and position of viable home ranges that can be maintained across a spatially heterogeneous area. We estimated parameters for this model from data on red-backed vole (*Myodes gapperi*) abundances in 31 boreal forest sites in Ontario, Canada. The home range model had considerably more support from these data than both non-spatial regression models based on the same original habitat variables and a mean-abundance null model. It had nearly equivalent support to a non-spatial regression model that, like the home range model, scaled an aggregate measure of habitat value from local associations with habitat resources. The home range and habitat-value regression models gave similar predictions for vole abundance under simulations of light- and moderate-intensity partial forest harvesting, but the home range model predicted lower abundances than the regression model under high-intensity disturbance. Empirical regression-based approaches for predicting species abundance may overlook processes that affect habitat use by individuals, and often extrapolate poorly to novel habitat conditions. Mechanistic home range models that can be parameterized against abundance data from different habitats permit appropriate scaling from individual- to population-level habitat relationships, and can potentially provide better insights into responses to disturbance.

## Introduction

Relationships between a species' abundance and its habitat are commonly described using statistical methods such as regression analysis, but resulting empirical models do not directly capture the processes involved in determining abundance, nor can they be reliably extrapolated outside the conditions for which data were collected. Alternatively, mechanistic approaches for modelling species abundance in different habitats can offer a powerful means of predicting population responses to habitat alteration [1], [2]. Towards this end, greater understanding and generality may be obtained by developing models that explicitly translate from individual-scale resource selection in heterogeneous environments, to the spatial distribution and abundance of a larger population [3]. This individual-to-population scaling can be achieved by formulating the process of home range establishment as an individual's optimization of trade-offs between resource acquisition, home range size, and overlap with conspecifics [4].

Various methods have been developed for predicting home range behaviour using location or movement data for individual animals (reviewed in [5]), with recent work seeking to link mechanistic models with resource selection analysis [6]. In a more general approach, Mitchell and Powell [7], [8] formulated home range models within an optimal patch-selection framework, then tested these against empirical data for American black bears (*Ursus americanus*). Their models describe individual home ranges as a spatially explicit collection of habitat patches that either maximize resource accrual per unit area or minimize the area required to meet a specified resource threshold, where the resource value of patches is determined by their inherent quality, their travel cost, and their use by other individuals. Buchmann et al. [9] have presented a similar approach, generalizing it to the community level to understand how home range size and species abundance scale with body mass. With a given set of parameters and a map describing the distribution of habitat quality, these home range models can be scaled up to predict a species' carrying capacities for different landscapes. However, while the models present important conceptual advances, their predictions for species abundance have not been calibrated against data at the population scale, nor have such predictions been compared with those derived from simpler alternative approaches.

Models for species abundance that scale from a collection of individual home ranges hold particular promise for spatially heterogeneous habitats that are subject to anthropogenic or natural disturbance. In many forest ecosystems, stands develop a high level of fine-scale heterogeneity as they reach late stages of development and are subject to gap-phase disturbance dynamics [10], [11], [12]. Such patterns of within-stand disturbance have, in turn, inspired stand management ideas focused on creating a variable distribution of gap sizes that aim to maintain the ecosystem functioning of old, complex stands [13]. Conversely, retention of residual trees in different spatial configurations is also important under clearcut harvesting for maintaining structural and biological legacies that persist following stand-replacing disturbances [14]. A home range modelling approach may be better suited to predicting the consequences of natural and induced heterogeneity in habitat structure than empirical models that presume conditions are relatively uniform within stands.

Here we developed a simple, mechanstic, and spatially explicit model of optimal home range establishment and used it to predict abundances of a common late-successional microtine rodent in North American boreal forests, the southern red-backed vole (*Myodes gapperi*). We estimated parameters for this model from live-trapping data in 31 sites with mapped habitat distributions, and compared its performance to non-spatial regression models relating vole abundance to either a series of stand-level habitat attributes, or to a metric for average habitat value derived from these attributes. To illustrate the utility of our approach, we then applied these home range and regression models to simulations of various partial harvesting scenarios and compared predictions of vole abundance between the different models.

## Methods

We previously developed an empirical model of fine-scale red-backed vole habitat associations for mixedwood forest stands in Ontario, Canada [15]. Using live-capture data from 30 managed (31–64 year old) and 10 fire-origin (82–156 year old) sites, our results indicated that within-stand locations of spring vole captures were associated with localized shrub cover, late-decay downed woody debris (DWD), shade-tolerant understory composition, and conifer-associated litter on the forest floor. Here, we used these results as a basis for mapping local habitat resources at a 15-m resolution within 31 of these sites (21 managed and 10 fire-origin for which live-trapping data were available over two years; the remaining sites were subjected to experimental manipulations between the two trapping years). We then used estimated vole abundances within these sites to parameterize a spatially explicit optimization model of home range establishment that predicts vole population density, as described below. Along with the description of the model that follows, we have created a spreadsheet that provides a detailed walkthrough of the model calculations for one example site (see Appendix S1).

### Mapping Local Habitat Value

The habitat model in [15] describes how red-backed voles exhibit increased use of localities where particular habitat resources are abundant relative to the average habitat conditions across 1.4 ha sites:
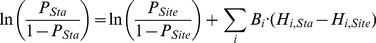
(1)where the response variable, *P_Sta_*, is the probability of a new capture at an individual live-trap station. *P_Site_* is the new capture frequency for the site as a whole. For each of *i* habitat predictor variables, *H_i,Sta_* represents mean value within 26 m of the trap station, and *H_i,Site_* is the overall site mean. The predictors are related to the response by the coefficients *B_i_*, which could either (1) take on a constant value among sites (*B_i_ = b_i,0_*); or (2) vary as a logistic function of the site-level availability of that habitat feature:
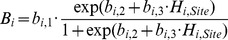
(2)where all subscripted instances of *b* are estimated parameters for the existing habitat model. The logistic functional response (eqn 2) represented situations where associations with local habitat features were conditional on their availability [16], as may occur if the habitat features are not limiting in all sites. Voles showed consistent associations with shade and substrate-related features across the observed range of sites (case 1 above), but were only associated with shrub cover and late-decay DWD in sites where these features were relatively sparse (case 2).

**Figure 1:**
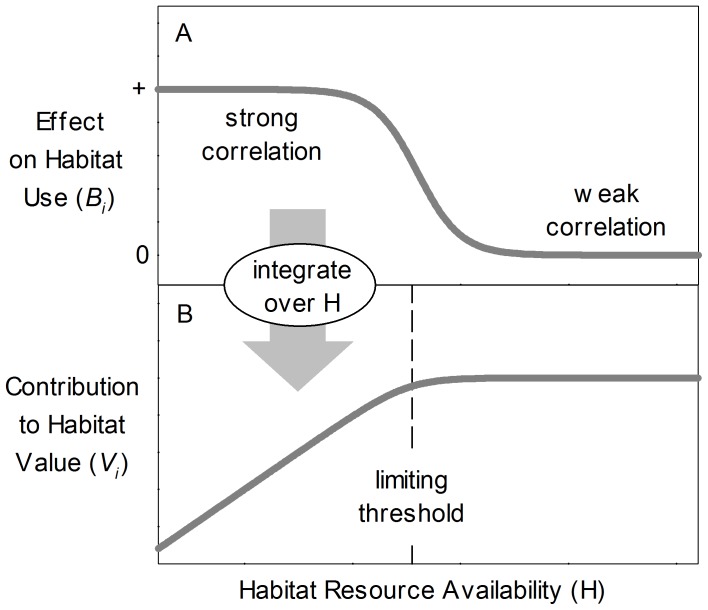
Derivation of habitat value from effects on relative habitat use. A species may show strong associations with the availability of a given habitat resource in areas where the resource is in limited supply, but not in areas where the resource's availability exceeds the species' requirements (A). When these conditional effects on habitat use are integrated over the resource's availability, the inferred value of the habitat is characterized by a threshold relationship with resource availability (B).

To calculate the value of local habitat resources for our new home range model, it was necessary to translate the relative effects of these habitat features within sites to an absolute measure that could be compared among sites. To do so, we first integrated each feature's per-unit effect on the probability of local occurrence:
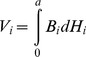
(3)where *V_i_* represents the contribution of amount *a* of habitat resource *H_i_* to local vole habitat value. Note that the coefficients *B_i_* in eq. 1 express rates of change in (logit of) occurrence probability with respect to local habitat resources (dLogit(*P_Sta_*)/d*H_i,Sta_*), and so each *V_i_* expresses the cumulative positive effects of a given resource on habitat use (Fig. 1). These cumulative measures of local habitat value reach an upper limit if voles are no longer associated with a resource where it is highly abundant.

We next calculated overall local habitat value (*V*) for all habitat resources as:
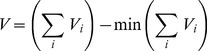
(4)where the summations are across the four habitat terms (shrub cover, DWD, shade, and substrate), and min(∑*V_i_*) represents the lowest-value local habitat found in the data used to parameterize the model. Given a mapped distribution of *V* across a site, we sought to estimate the maximum number of viable red-backed vole home ranges that could be established there.

### Home Range Model Description

**Figure 2:**
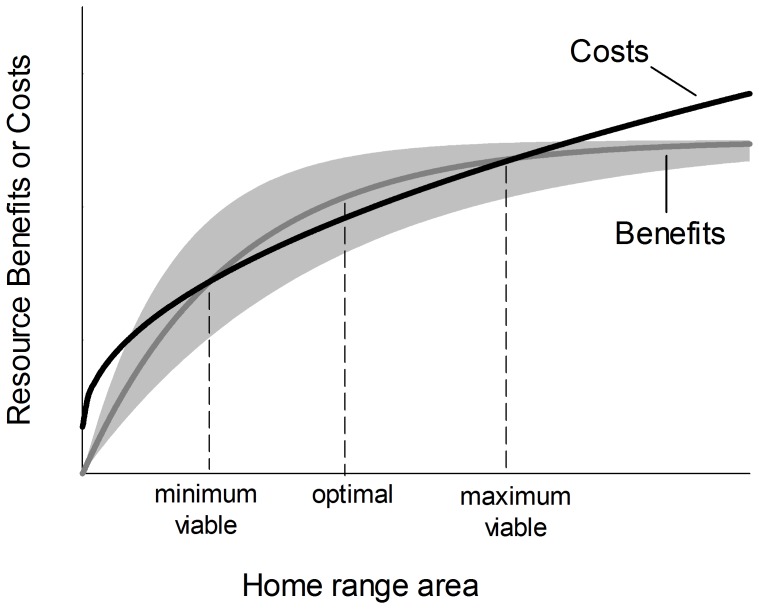
Representation of the benefits and costs of maintaining home ranges of different sizes. Home ranges where benefits are greater than or equal to costs in a given habitat are considered viable, and the home range with the greatest difference between benefits and costs is considered optimal for that habitat. The rate at which home range benefits accrue depends on both habitat value and overlap with other individuals, producing a range of possible curves (shaded region) and differences in the minimum, maximum, and optimal home range size among habitats.

Our home range model estimates the number, size, and position of viable home ranges that can be established in a given site, where viability is defined by a positive net balance between expected access to habitat resources and both fixed and area-dependent home range costs (Fig. 2). For each individual, a home range is represented by a contiguous set of at least four 15-m resolution cells (opposite edges were considered adjacent to one another to prevent partial home ranges from extending outside the grid of cells). All habitat cells were available for individuals to include in their home range, but the value of each cell varied inversely with the number of home ranges in which it was included. The resource benefits derived from a given home range scaled with the total habitat value of cells encompassed, asymptotically approaching an upper bound where additional resources provided no incremental value (Fig. 2). Accordingly, home range benefits (*HR_B_*) were calculated as:
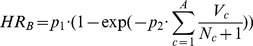
(5)where *A* is the number of cells included in the home range, *V_c_* is the habitat value of cell *c*, *N_c_* is the number of other home ranges that overlap cell *c*, and *p_1_* and *p_2_* are free parameters that determine the shape of the curve. The costs (*HR_C_*) associated with a given home range were represented by a fixed constant, expressing basal metabolic demands to be met through resource acquisition, plus the square root of home range area, expressing the costs of travel and exposure to predation (Fig. 2):
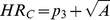
(6)where *p_3_* is a free parameter representing area-independent home range costs. These fixed costs determine the minimum resource benefits that need to be accrued, before accounting for additional costs of home range size. The net value (*HR_NV_*) of a given home range, measured in arbitrary units related to area, was then calculated as the difference between benefits and costs:
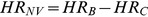
(7)


This formulation enabled us to estimate the value of a given home range as a function of the quality of habitat resources it encompasses, overlap with other individuals, and total size. Home ranges with a positive value for *HR_NV_* are considered to be viable, whereas those for which *HR_NV_*<0 are not. As the number of home ranges increases on a site, the average *HR_NV_* decreases because individuals are forced to partition resources in high-quality habitat patches, to maintain smaller home ranges with access to fewer resources, or both. Therefore, there is a limit to the number of viable home ranges that can be maintained at a given site, which depends on the local distribution of habitat resources.

We applied this model to estimate the potential number of home ranges that can be maintained in different sites using the following procedure. Starting with one home range of a fixed size and shape, we found the initial position that maximized *HR_NV_*. We then let this home range expand or contract by one or two cells, or remain static, such that the new home range had the highest *HR_NV_* from among those considered. This step was repeated until no further improvements in *HR_NV_* were possible. If the resulting home range had *HR_NV_*>0, we then added another home range and repeated the process, adding or removing up to two cells from each of the home ranges in turn and iterating until none could improve in *HR_NV_*. If one or more home ranges had *HR_NV_*<0, we re-established home ranges from new initial conditions to again see if a viable set could be obtained. For a given set of model parameters (*p1*, *p2*, *p3*), we estimated resident vole abundance within a site as the maximum number of home ranges that could be established where *HR_NV_*>0 for each.

### Field Data and Parameter Estimation

Our model purposely simplifies aspects of home range behaviour in order to minimize the number of free parameters that need to be estimated. While this necessarily sacrifices some degree of biological realism, it enables the use of powerful inverse modelling techniques [17] to parameterize the model solely from data on species abundances in different habitats. Inverse modelling estimates the home range parameter values that maximize the fit of emergent population densities (as predicted by the resulting model) to observations. The approach thereby allows the home range model to be quantitatively compared with alternative empirical models derived from the same data.

We estimated the values for *p_1_*, *p_2_*, and *p_3_* that provided the best fit to vole abundances in 31 boreal mixedwood sites near Kapuskasing, Ontario, Canada (49°25′N, 82°25′W). At each of these sites we live-trapped red-backed voles in the spring (7 May–4 June) of 2006 and 2007 (University of Toronto Animal Use Protocol 20005744, 20006270, and 20006824), and measured spatially-explicit habitat variables relevant to the model of local habitat value (eq. 1–4; Table 1). Further details on the sites, habitat measurements, and live-trapping procedures used are provided in [15]. Although this is largely the same dataset used to build the fine-scale habitat model, there was no circularity in the parameterization of the home range model because the earlier work used capture locations within sites as a response variable while controlling for site-level capture frequency. Indeed, the mean habitat effect on capture probability was approximately zero for each site; across sites, there was virtually no correlation with the final predicted number of home ranges (*r* = −0.10). Parameter estimation for the home range model did not use any information on capture locations within sites.

**Table 1:** Summary of methodology for quantifying vole abundance and local habitat value in 31 boreal mixedwood sites in Ontario, Canada, 2006–2007.

Quantity	Field sampling methods	Model form
Vole abundance	Live-trapping over 3 consecutive nights using 49 traps set in 7×7 grid at 15 m intervals.	Average no. individuals, adjusted for detectability as described in text.
Shrub cover	Counts of stems ≥1 cm height within 221 1-m^2^ quadrats set at 5 m intervals.	Weighted sum of counts in four classes, transformed to have an upper asymptote.
Downed woody debris	Line-intersect sampling along 14 120-m transects.	Sum of diameters raised to an estimated exponent, per unit of transect.
Shade	Same as shrubs above.	Gradient from detrended correspondence analysis (DCA) on species composition.
Substrate	Percent cover of four ground cover types in 221 1-m^2^ quadrats set at 5 m spacing.	Weighted sum of ground cover types.

Live-trapping was carried out when populations were near their annual minimum and individuals would have been able to establish home ranges in the best local habitat present. Dispersal rates among sexually mature red-backed voles are reportedly very low [18], so we expect that captures generally sampled from local resident populations.

Variation in animal detectability (probability of an individual's capture given that it is present) can affect estimates of population size [19], and so we sought to correct the number of individuals captured for possible year- and site-specific differences in detectability. For each year and site, the log-likelihood (*LL*) for the observed sequence of captures of new individuals ({*x_1_, x_2_, x_3_*}) over three days was:
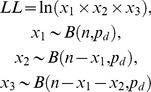
(8)where *n* is the estimated total number of voles at the site, *p_d_* is their estimated daily detectability, and *B*(·) is probability density from a binomial distribution. We estimated the values for *n* and *p_d_* that maximized the total log-likelihood across both years and all sites in models where *p_d_* was either year-specific, site-specific, year- and site-specific, or a global constant. We found the greatest support for a model in which estimated detectability varied between years (*p_d,2006_* = 0.63; *p_d,2007_* = 0.42), but not among different sites (ΔAIC_C_ = 10.9 for next best model). Using this detectability model we estimate that, over three trapping days, we captured 95% and 81% of voles present at each site in 2006 and 2007, respectively. For each of the 31 sites, we divided the number of individuals captured each year by their year-specific detectability over three days, averaged these values across both years, and rounded this average to the nearest integer. The result was used as our measure of vole abundance at each site.

Assuming a Poisson error distribution, we compared observed (corrected for detectability) and predicted vole abundances to calculate the likelihood of a given set of parameters in the home range model. We used a simulated annealing algorithm [20] to maximize the sum of the log-likelihood of all observations across the dataset and estimate best-fit values for *p_1_*, *p_2_*, and *p_3_* [21].

### Alternative Models

We separately fit a constant and three Poisson regression models to this same dataset to evaluate the ability of the home range model to predict site-level vole abundances. In the first regression model, we sought to assess whether our home range model performed better than a model that used the same predictor variable for habitat value, but that disregarded its spatial variability within sites. This model predicted vole abundance (*Y*) by a log-linear function of average habitat value (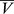) within each site:
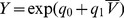
(9)where *q_0_* and *q_1_* are regression coefficients.

In the second and third regression models, we sought to assess whether our home range model and habitat value metric performed better than an empirical model constructed using site averages for the original habitat resources (H_i, Site_; equation 1). Although we originally started with four habitat variables, we decided to first use only two of these in order to restrict the ratio of parameters to data and lessen the risk of overfitting. After some preliminary comparisons, we found that the best two-variable habitat model included the effects of shrub cover (H_Shr, Site_) and shade-tolerant understory composition (H_Sha, Site_):
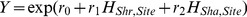
(10)where *r_0_*, *r_1_*, and *r_2_* are regression coefficients. For completeness, we also present results for the equivalent regression model that included all four of the original habitat resources (*r_3_* and *r_4_* are regression coefficients for DWD and substrate, respectively).

Lastly, for the constant model we predicted vole abundance as the mean among sites (*k*). This represented a null model for no relationship between vole abundance and our habitat metrics:
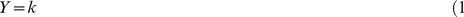
(11)


We compared the home range, regression, and constant models using Akaike's Information Criterion corrected for small sample sizes (AIC_c_)

### Application to Partial Harvesting Scenarios

We next applied the home range and regression models to simulated forest stands in which the intensity and spatial pattern of recent (5-year) harvesting were varied systematically. These scenarios varied in the magnitude of habitat alteration resulting from harvesting and in the degree of spatial heterogeneity induced. As such, the scenarios provided a range of conditions through which expected population-level responses under the non-spatial regression models and the spatial home range model could be compared.

**Figure 3:**
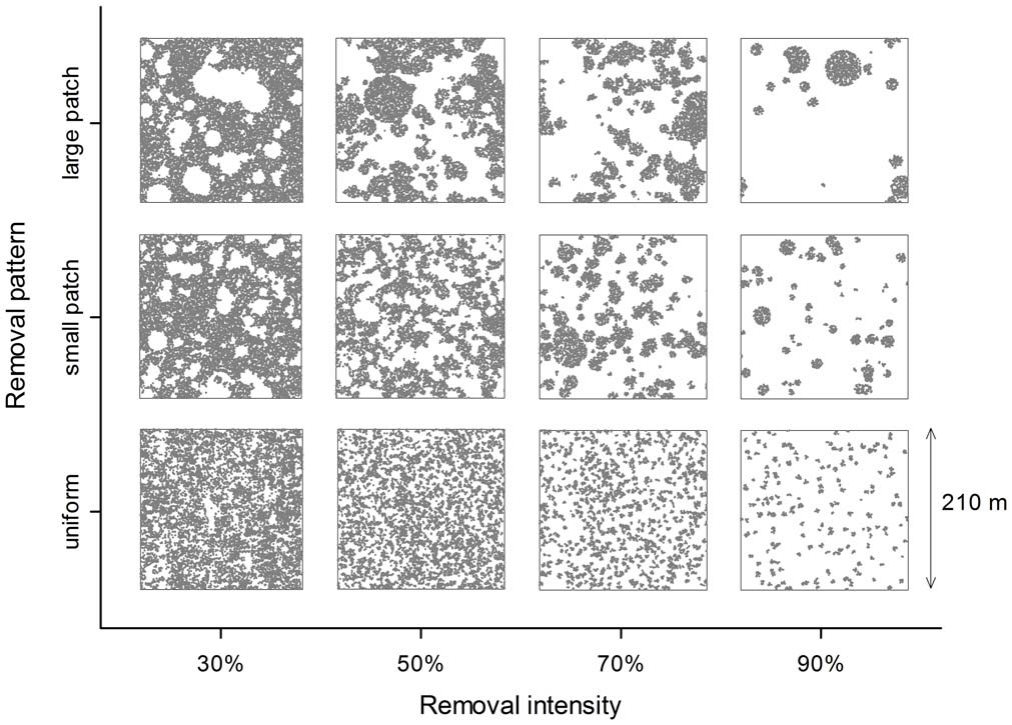
Stem maps depicting examples of simulated partial harvesting. Simulations included four levels of intensity (increasing from left to right) and three levels of aggregation (increasing from bottom to top).

**Figure 4:**
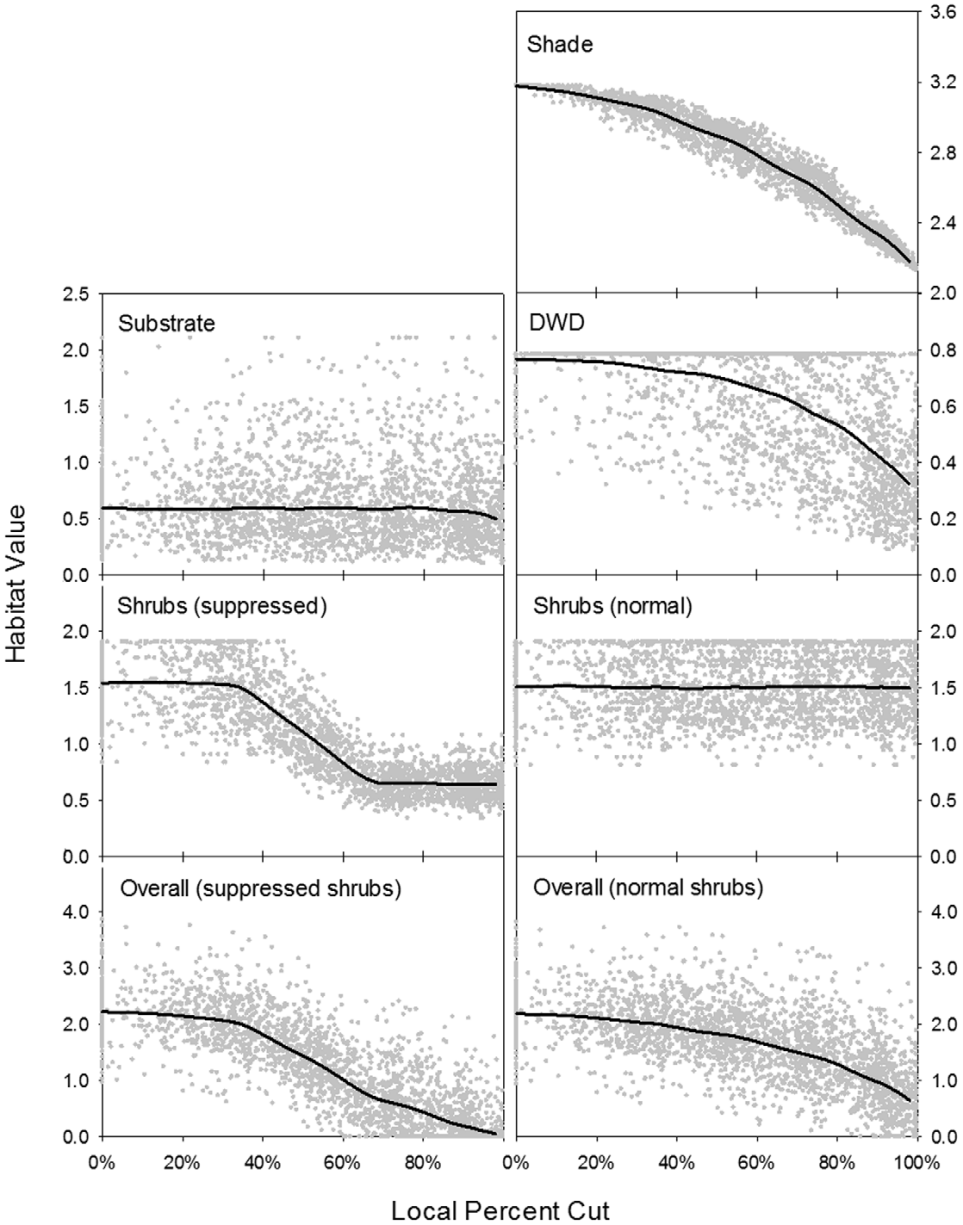
Simulated value of habitat components as a function of local intensity of partial harvesting. Shrub cover and overall habitat value are presented for both the normal and suppressed shrub development scenarios (see text for details). Solid black lines indicate habitat trends derived from LOESS regression.

We used the individual-based stand dynamics model SORTIE-ND [22], [23], to generate spatially explicit 210×210 m stem maps for relatively old boreal mixedwood stands. Fifteen replicate stands were initially grown for 160 years from bare ground conditions, by which time, like the field sites, their composition was approximately equally divided between deciduous and coniferous species. We simulated partial harvesting in each stand at four levels of harvest intensity (30%, 50%, 70%, 90% basal area removal) and, for each, three levels of harvest pattern (uniform, small-patch, large-patch). To achieve different spatial patterns of harvesting, we varied the size distribution of groups of trees to remove and retain by drawing random deviates from a lognormal distribution: with the mode held constant, we simulated greater degrees of harvest aggregation by systematically increasing the variance in group area (Fig. 3). As we explain below, we used these stem maps together with published results describing effects of partial harvesting on stand structure to derive expected vole habitat value in 15×15 m cells within the simulated stands, both before and five years after harvesting (Fig. 4).

The only vole habitat feature that could be gleaned directly from the model's output was shade. SORTIE-ND computes the percent of total sunlight (gap light index, GLI) transmitted through tree crowns at a given point in space. We sought to translate GLI into a measure of shade represented by an ordination of understory shrub composition in the habitat model [15]. To do so, we calculated each of the five tree species' optimal GLI level (i.e., where each had the fastest growth relative to the others). We then regressed these values against ordination-based mean shade values of 1-m^2^ plots in which saplings of each species were present in the actual stands. The resulting relationship (Shade = 3.6802–0.0132·GLI) showed a strong fit (R^2^ = 0.95) across the five species, and enabled us to predict shade habitat values within 15×15 m cells of each simulated stand.

Substrate habitat largely distinguished coniferous from deciduous litter on the forest floor. We used a relationship between this habitat term and proportion coniferous basal area (PCBA) from empirical data to estimate vole substrate value in cells of the simulated stands (Substrate = 0.1193+0.1816·PCBA; R^2^ = 0.17). Residuals from this relationship showed a lognormal distribution and strong short-distance autocorrelation. To add realistic spatial variability to the simulated stands, we applied lognormally-distributed scatter to the estimated substrate values (using the average standard deviation from within real unmanaged mixedwood stands) and re-arranged the cell scatter values to match observed autocorrelation patterns.

We used published studies from nearby areas to estimate the effects of partial harvesting on DWD and shrub cover. In aspen-dominated stands of northwestern Quebec, Harvey and Brais [24] found that well-decomposed DWD was less abundant in 1/3- and 2/3-removal cuts than in control stands 6 years after harvesting. To apply these results to the simulations, we calculated their observed decreases in DWD after partial harvesting relative to unharvested stands, and multiplied these values by the average amount of late-decay DWD in unmanaged stands. The resulting three points fell on a straight line (DWD = 2.2035–1.8336·PC; R^2^ = 1.00) describing a decrease in DWD with increasing proportion of basal area cut (PC). As with substrate, we applied lognormally-distributed and autocorrelated scatter to these values based on observed variability in DWD within unmanaged stands.

Studies across the boreal forest have generally failed to identify an effect of partial harvesting on shrub cover [25], [26], [27], although partial harvesting has had a strong negative effect on shrub cover in the Pacific Northwest region [28]. However, chemical tending practices sometimes used to promote successful regeneration in partially harvested stands have been found to reduce shrub cover by approximately 60% [25]. We therefore developed two scenarios for post-harvest shrub development. In the ‘normal’ shrub cover scenario, shrub cover was unaffected by harvesting and was assigned the mean value from unharvested stands. In the ‘suppressed’ shrub cover scenario, shrub cover was unaffected in cells where the percent of basal area cut was less than 33%, was reduced by an average of approximately 60% where the percent cut exceeded 67%, and decreased linearly between these levels at cutting intensities of 33–67%. This scenario was used to examine the effects of post-harvest herbicide application, and to compare predictions of the home range model where the contrast between lightly harvested and intensely harvested cells was enhanced through reductions in shrub cover. Again, we applied lognormally-distributed and autocorrelated scatter based on observed variability within unmanaged stands (Fig. 4).

## Results

### Model Parameterization

Across the 31 1.4 ha sites, mean (±SD) spring red-backed vole abundance was estimated to be 7.8 (±4.5) individuals. The home range model explained a modest amount of variation in vole abundance among these sites (R^2^ = 0.25), with predicted abundances ranging from 3–10 individuals. Comparisons of AIC_c_ values with those for constant and regression models (Table 2) indicated that the home range model fit the data much better than a constant value (ΔAIC_c_ = 12.0), and substantially better than regression models that used original habitat variables (ΔAIC_c_ = 5.9, 8.3). Given their low degree of support from the data, the constant and original-habitat-variable models received no further consideration.

**Table 2:** Comparison of the fit of home range, regression, and constant models to the 2006–2007 spring abundances of red-backed voles across 31 boreal mixedwood sites in Ontario, Canada.

Model	No. Parameters	Parameter Values	Log-likelihood	ΔAIC_c_	R^2^
Home range	3	*p_1_* = 8.108, *p_2_* = 0.222, *p_3_* = 4.150	−87.95	0.0	0.25
Regression against derived habitat value	2	*q_0_* = 0.856, *q_1_* = 0.711	−89.21	0.1	0.19
Regression against original shrub and shade variables	3	r_0_ = −0.473, r_1_ = 2.621, r_2_ = 0.643	−90.90	5.9	0.13
Regression against all original habitat variables	5	r_0_ = −1.575, r_1_ = 3.704, r_2_ = 0.778, r_3_ = 0.144, r_4_ = 1.253	−89.34	8.3	0.18
Constant	1	*k* = 7.774	−96.33	12.0	0.00

**Figure 5:**
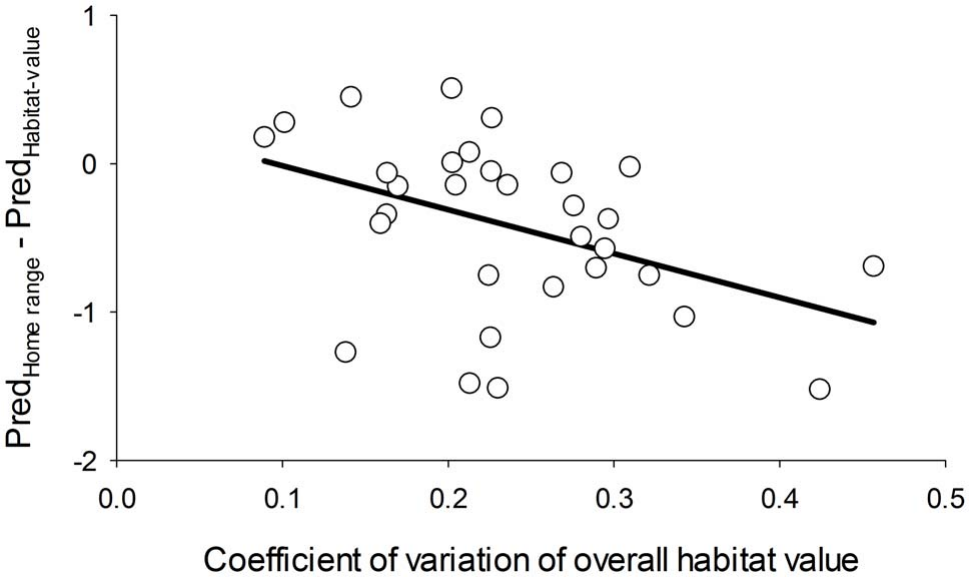
Difference between home range and habitat-value model predictions plotted against within-stand variation in habitat value. The solid black line represents the linear trend in the relationship.

The home range model had virtually the same support from the data as a regression model that used our derived metric for habitat value (ΔAIC_c_ = 0.1), though the latter model explained less variation in the data (R^2^ = 0.19 with one fewer parameter). Although predicted abundances under the home range model and habitat-value regression model were similar, the home range model tended to predict a greater number of voles than the regression model in sites with more uniform local habitat values, and fewer voles in sites that had more varied local habitat (Fig. 5).

Mean (±SD) home range size was 7.3 (±1.2) grid cells, or 0.16 (±0.03) ha. There was two-fold variation in the size of modelled home ranges, from 5 to 10 grid cells (0.11 to 0.23 ha). Among the 1519 grid cells in all 31 sites, 72% were included in one home range, 18% were included in two home ranges, and 1% were included in three home ranges. Nine percent of grid cells were not included in any of the optimized home ranges. As the estimated number of home ranges increased, individual home ranges both tended to become smaller (*r* = −0.34) and exhibited greater overlap with one another (*r* = 0.71).

### Partial Harvest Simulations

**Figure 6:**
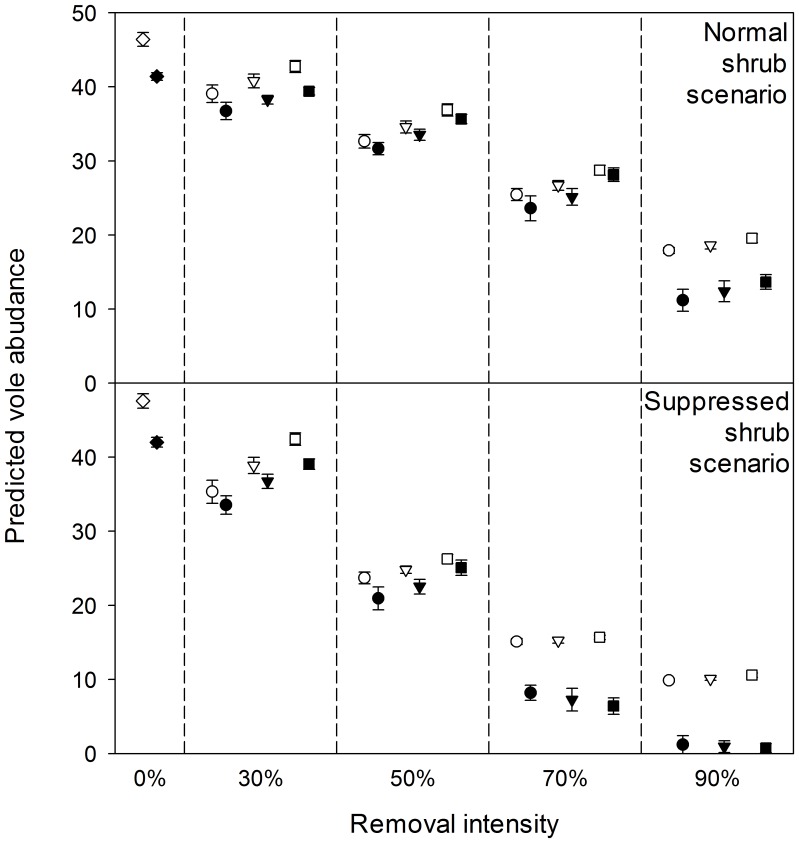
Comparisons of predicted red-backed vole abundances (±SD) in various simulated partial harvesting scenarios. Predictions from both the habitat-value regression (open symbols) and home range (filled symbols) models are shown. Circles, triangles, and squares represent large-patch, small-patch, and uniform harvest patterns, respectively. Diamonds represent unharvested stands.

Applying the home range and habitat-value regression models to simulations of habitat changes through partial harvesting, red-backed voles were predicted to decrease in abundance across a gradient in harvest intensity. Voles were fairly resilient to moderate levels of basal area removal in the normal shrub cover scenarios, but showed markedly greater decreases where post-harvest shrub cover was suppressed through herbicide application (Fig. 6). Although the spatial pattern of harvesting had a minor effect on abundance compared to both harvest intensity and shrub suppression, voles reached somewhat higher abundances under uniform vs. large-patch retention patterns at low intensities of disturbance.

Under the home range model, decreases in the abundance of voles were mostly attributable to lower degrees of home range overlap (Table 3), and to an increasing area not included in any home ranges. Mean home range size varied by only 13% among the partial harvest scenarios (Table 3).

**Table 3:** Mean habitat value, mean home range size, and home range overlap under the home range model for mixedwood field sites in Ontario, Canada, and for simulations of unharvested and partially harvested stands.

	Normal shrub development			Suppressed shrub development		
	Mean habitat value (±sd) *	Mean home range size (±sd) †	Home range overlap (%) ‡	Mean habitat value (±sd) *	Mean home range size (±sd) †	Home range overlap (%) ‡
Field sites	1.64±0.35	7.3±1.2	35%	-	-	-
Unharvested	2.24±0.47	8.3±1.1	82%	-	-	-
30% removal						
Large-patch	2.00±0.53	7.9±1.1	67%	1.86±0.67	8.2±1.1	70%
Small-patch	2.06±0.48	8.0±1.1	70%	1.99±0.53	8.1±1.1	69%
Uniform	2.13±0.48	8.0±1.1	73%	2.12±0.48	8.0±1.2	72%
50% removal						
Large-patch	1.75±0.55	8.0±1.1	54%	1.30±0.74	8.1±1.1	49%
Small-patch	1.83±0.52	7.9±1.1	57%	1.36±0.64	8.2±1.0	44%
Uniform	1.92±0.49	7.9±1.1	61%	1.44±0.50	8.1±1.0	33%
70% removal						
Large-patch	1.40±0.59	8.0±1.1	39%	0.70±0.65	8.3±1.1	35%
Small-patch	1.47±0.56	8.0±1.0	41%	0.69±0.56	8.3±0.9	23%
Uniform	1.57±0.51	7.9±1.0	42%	0.72±0.45	8.4±0.8	11%
90% removal						
Large-patch	0.91±0.55	8.2±0.9	23%	0.25±0.43	7.3±1.7	19%
Small-patch	0.96±0.45	8.3±1.0	22%	0.25±0.37	7.4±1.2	8%
Uniform	1.03±0.50	8.3±0.9	18%	0.26±0.35	7.8±1.3	0%

* Mean±SD of vole habitat value per cell (eq. 3 and 4).

† Home range size is given in units of 15×15 m grid cells.

‡ Mean percentage of each home range that is shared with one or more other home ranges.

The home range and habitat-value regression models gave similar predictions for harvest types that induced small and moderate degrees of habitat change (30–70% removal with normal shrub cover and 30–50% removal with suppressed shrub cover). However, as habitat became more heavily degraded by high-intensity silvicultural practices, the home range model predicted greater decreases in abundance than the habitat-value regression model.

## Discussion

Habitat selection at local-patch and home-range scales involve decisions where individuals must assess how fitness is affected by the quality of habitat, interference from conspecifics, travel, and potentially a multitude of other factors [29]. Selection of habitat features is typically scale-dependent, such that features that describe good habitat at fine scales may not do so at broader ones [30]. Theoretical developments in habitat selection have sought to integrate these various processes and scales through frameworks that generate predictions of population density from simple underlying mechanisms [3], [31], [32], [33]. In particular, the classical concept of an ideal free distribution [34] has proved highly influential in explaining how individuals distribute themselves among habitat patches when the fitness value of each patch depends on both its inherent quality and the number of individuals that occupy it.

Our model of home range establishment operates at an individual level to estimate use of space as a trade-off between habitat value, overlap with conspecifics, and home range size. It accounts for partitioning of resources among individuals that share a given habitat patch and, following the principles of an ideal free distribution, promotes dispersion through density-dependent selection of patches according to their fitness value. By simulating iterative changes in the positions of a set of individual home ranges, the model estimates the maximum number of feasible home ranges that can be established within a heterogeneous area when individuals optimize the difference between resource benefits and costs.

### Evaluation of the Home Range Model

We employed inverse modelling, whereby data at a given scale (e.g., population) are used to make inferences on processes operating at the next-lower scale (e.g., individual home ranges), to maximize the model's fit to observed vole abundances across a number of sites. Unlike many other spatially explicit home range models, this approach does not require observations of the location or movement of individuals. In addition, calibration of the home range model from vole abundances enabled direct comparison to three regression models based on the same data. Naïve application of the original terms that explained local habitat use did not predict stand-level patterns of abundance well (Table 2). It seems, therefore, that the method by which we translated predictors of local habitat use into an aggregate measure of habitat value (eqns 3, 4) was important to understanding how they affect home range establishment and population density. In particular, limiting thresholds (Fig. 1) may have a key role in determining habitat relationships [35], but such complexities are generally not incorporated into empirical habitat analyses built using linear models.

The home range model received nearly equivalent support to (but had somewhat greater explanatory power than) a regression model where the individual effects of each variable were incorporated into an aggregate measure of habitat value, then averaged across each site (Table 2). Although predicted abundances under the home range and habitat-value regression models were highly concordant, the home range model tended to predict lower vole abundances than the habitat-value regression model in stands with more heterogeneous habitat (Fig. 5). Within-stand variability in vole habitat value was fairly low in the field sites (mean CV = 24%), so it is interesting that differences between the two models, albeit modest ones, arose from this heterogeneity. One might expect such differences to be more pronounced in sites subjected to variable intensities of disturbance, which we indeed found in certain simulations of partially harvested stands (discussed further below).

Unlike the regression models, our home range model generates a number of further predictions concerning vole home range size, location, and overlap. Reported home range sizes for red-backed voles range from 0.09–0.5 ha [18], and the predicted mean of 0.16 ha was within this range. Although the model produced two-fold variation in home range size, mean home range size among sites was only weakly related to the density of individuals. Field observations have shown that mean home range sizes of mature females vary little within habitat types, and are independent of population density [18]. The model further predicted that habitat patches would commonly be included in one home range, would occasionally overlap two home ranges, and would rarely be used by three or more individuals. Mature female red-backed voles maintain home ranges with little overlap during the breeding season [18], but home ranges of males and immature females may overlap with each other and with those of mature females [36]. These patterns are broadly consistent with the degree of overlap observed in the simulated home ranges.

### Simulations of Partially Harvested Stands

Red-backed voles tend to increase in abundance with stand age, reaching their highest densities in mature and old stands with complex structural features [37]. They are tolerant of light- and moderate-intensity partial harvesting, but often exhibit decreased abundance where harvesting removes more than about two-thirds of stand basal area (reviewed in [38]). These changes appear to be attributable to the alteration of important habitat conditions such as protective cover and cool, moist microclimates. Voles exhibit less marked decreases in abundance in harvested stands where such features are maintained [39], [40].

As a model evaluation exercise, we compared our simulation results to these general patterns in red-backed vole responses to partial forest harvesting. The simulations were in agreement with previous empirical results: with normal shrub development, estimated abundances of red-backed voles decreased by <30% at harvest intensities of up to 50% removal, but then decreased by about 60–70% at 90% removal (Fig. 6). Our simulations for post-harvest shrub suppression appropriately reflected the strong adverse effects such practices are known to have on this species [41].

At low removal intensities, both models predicted that dispersed retention could support somewhat higher vole abundances because habitat within these stands was better shaded, on average, than in stands subjected to aggregated harvesting. In highly degraded habitat scenarios the effect was offset by greater average shrub cover under aggregated harvesting, leading to little difference in vole abundance among harvest patterns (Fig. 6). The home range model further predicted that when harvesting induced spatial heterogeneity in local habitat, voles tended to establish home ranges within less-disturbed areas, particularly as the overall intensity of disturbance increased. In an experimental comparison of different harvest patterns, there were no significant differences in small mammal abundances between dispersed and aggregated retention treatments at 75%, 40%, or 15% retention [40]. However, 70% of red-backed vole captures in the aggregated retention treatments occurred in the unharvested portion of those stands [42].

Model predictions in the partial harvest simulations diverged in scenarios with high degrees of habitat degradation, with the home range model predicting sharper decreases in abundance than the habitat-value regression model (Fig. 6). Whereas the regression model assumed that log-abundance was linearly related to habitat value, the home range model supposed a lower threshold below which voles could not establish viable home ranges. Many species show abrupt decreases in habitat use beyond a given level of habitat alteration, even after accounting for local habitat suitability [43]. Such effects cannot be detected by regression models when extrapolating outside the range of data used for their development. However, if a mechanistic model captures underlying processes which govern habitat use, and these processes are invariant to habitat alteration, then we might place greater confidence in its predictions under novel habitat conditions. Although we cannot verify that our home range model meets these conditions, its predictions suggest one potential mechanism for non-linear responses to habitat alteration that could be evaluated in future research.

### Application in New Contexts

Although the home range model generated valuable insights for both the original calibration data and simulated partial harvesting scenarios, its extension to new landscapes, habitats, and populations remains untested. It should also be noted that although the home range model had a better fit to the data than any of the alternative models considered, it was still unable to explain most of the variation in vole abundance among sites. Independent testing of the model is needed to verify its performance under new habitat conditions, as well as to assess how its parameters vary, for example, with inter-annual fluctuations in population density. The simulations presented here explore how the model may behave under varying degrees of habitat alteration; these results provide specific predictions that we believe should be tested against real data from partially harvested boreal mixedwood stands [44].

We calibrated the home range model on top of an existing model that describes the fine-scale habitat assocations of red-backed voles within these stands. This may seem to impose a strong restriction on its application to new contexts, but other approaches could be used as well. One possibility would be to generate a habitat value map based on a resource selection function [45] developed from other available data appropriate to a given context. A second possibility would be to use inverse modelling to infer parameters for a model describing habitat value along with the parameters of the home range model, using only a set of abundance data and spatially-explicit habitat variables. Although this approach does not require *a priori* information on habitat quality, it would require a larger amount of abundance data because the parameters for habitat value are not contrained by any lower-level data on habitat use.

### Conclusions

Optimal home range models such as the one developed here apply a mechanistic biological foundation to the problem of estimating species abundances within heterogeneous habitats. With empirical data, they can test predictions concerning behavioural strategies for home range establishment and spatial dispersion [8]. With simulated data, they can generate predictions of how a species may respond to habitat alteration scenarios with varying degrees of spatial heterogeneity. For example, individuals have been found to vary home range size in response to the loss and fragmentation of good habitat patches [46], as well as in response to edge-related habitat conditions created by forest disturbance [47]. Mechanistic home range models like ours can explain how such changes in individual behavior as a result of habitat alteration might scale up to population-level impacts.

Simple phenomenological models for predicting species abundance may overlook processes that affect habitat use by individual animals. As ecologists increasingly recognize the importance of heterogeneity within forest ecosystems, mechanistic models that appropriately scale from individual- to population-level can have an important role in understanding species-habitat relationships under natural and anthropogenic disturbances.

## Supporting Information

Appendix S1 **Walkthrough of home range model calculations for an example site.**
(ZIP)
